# A scenario-based volcanic hazard assessment for the Mount Meager Volcanic Complex, British Columbia

**DOI:** 10.1186/s13617-022-00114-1

**Published:** 2022-04-29

**Authors:** Rachel Warwick, Glyn Williams-Jones, Melanie Kelman, Jeffrey Witter

**Affiliations:** 1 Centre for Natural Hazards Research, Department of Earth Sciences, Simon Fraser University, 8888 University Dr., Burnaby, BC V5A 1S6 Canada; 2 Geological Survey of Canada, 1500-605 Robson St., Vancouver, BC V6B 5J3 Canada; 3 Innovate Geothermal Ltd., 104-445 West 2nd Ave., Vancouver, BC V5Y 0E8 Canada

**Keywords:** Mount Meager, Hazard scenarios, Pyroclastic density currents, Lahars, Tephra fall

## Abstract

The Mount Meager Volcanic Complex (Mount Meager) is a glacier-clad stratovolcanic system in southwestern British Columbia which last erupted over 2400 years ago (VEI 4). While this is Canada’s most recent major explosive eruption, most past research on Mount Meager has focused on its numerous and large volume landslides and thus the volcanic hazard characteristics remain understudied. Here we present a suite of scenario-based hazard maps and an assessment addressing a range of potential future explosive eruptions and associated hazards. In order to overcome limited knowledge of the eruptive history, numerical models have been used to simulate the primary syneruptive hazards of concern (dome-collapse pyroclastic density currents, lahars and tephra fallout) largely utilizing eruption parameters from analogous volcanoes, i.e., glacier-clad stratovolcanoes in a subduction zone setting. This study provides a framework for similar volcanic hazard studies where geologic data is limited, funds are minimal, and access is difficult. Furthermore, this sets the stage for recognizing volcanic hazards in the Canadian landscape, providing a resource to prepare for and mitigate potential impacts well in advance of a crisis situation.

## Introduction

Explosive volcanic eruptions are often multi-hazard events and can impact proximal to distal regions around the volcano over various timescales. Volcanic hazard assessments and maps provide an essential resource for communicating about complex volcanic phenomena, and informing emergency management and mitigation plans. Many volcanic hazard assessments rely heavily on well-constrained eruptive history parameters (mapped and/or eye-witness accounts) and, where possible, monitoring campaigns (Calder et al. 2015).

Geology-based volcanic hazard maps, based on the mapped distribution of past events, are the most common type of hazard map. However, modelling-based assessments are increasingly being generated with the advent of improved software, computational performance, and a better understanding of the physical processes involved in the individual hazards. This type computationally simulates individual volcanic processes, often organized as scenarios (Calder et al. 2015). In cases where the geologic record may be insufficient to generate a geologically-based hazard map, the modelling-based approach may be particularly advantageous, as parameters may be taken from analogous volcanoes that have well-constrained eruptive histories. They may be applied deterministically (uses specific eruption parameters to characterise a scenario), probabilistically (uses probability to quantify hazards, includes variation of randomness), or as a hybrid (Marzocchi and Bebbington 2012; Rouwet et al. 2017; Ang et al. 2020).

The eruptive history of Canadian volcanoes is mostly unknown, and they have limited volcano-targeted monitoring. Mount Meager (*Qw’elqw’elústen* in the Líľwat language) located on the traditional territory of Líľwat Nation, is no exception to this. Only the most recent eruption (2360 calendar years B.P.) has been well studied (e.g., Hickson et al. 1999; Andrews et al. 2014). A second eruption has recently been investigated and dated, indicating that the complex has explosively erupted at least twice within the past 25,000 years (Russell et al. 2021). It is nevertheless a potentially active volcano with a dynamic fumarole field and multiple hot springs in the vicinity. Furthermore, due to its location in southwestern British Columbia near large population centres and critical infrastructure, Mount Meager has the potential for significant impacts. It has been semi-quantitatively assessed as the Canadian volcano that poses the greatest threat to people and infrastructure (Wilson and Kelman 2021). As such it warrants a volcanic hazard assessment to inform future monitoring and preparedness efforts.

Given the lack of detailed knowledge of eruption frequencies and eruptive characteristics, a deterministic approach is followed, organized as a scenario-based volcanic hazard analysis. Three plausible scenarios are considered that represent a range of reasonable eruption magnitudes that could define the next phase of explosive volcanism at Mount Meager with potentially destructive impact. The three hazards considered here are: dome-collapse pyroclastic density currents (PDCs), lahars (associated with the mixing of pyroclastic material and glacier melt) and tephra fall. These three particular hazards were included in this assessment as they are primary syneruptive in nature, have occurred during the last major eruption of Mount Meager (Stasiuk et al. 1996; Hickson et al. 1999) and caused some of the most destructive impacts in the aftermath of other explosive volcanic eruptions (in terms of fatalities in the case of lahars and PDCs, or being the most widespread product affecting social and economic activity in the case of tephra fallout) (Auker et al. 2013). Choosing to highlight and investigate these three volcanic hazards best informs emergency managers and stakeholders for emergency response plans and policy considerations. Secondary hazards associated with effusive volcanism and/or noneruptive hazards are not accounted for in this assessment.

Numerical modelling parameters are largely informed from documented eruptions of analogue volcanoes (i.e., glacier-clad stratovolcanoes with a history of explosive volcanism and associated with a subduction zone setting). This enables us to analyze the impacts of a future eruption (i.e., inundation footprint, timeframe, topographical modifications) in the absence of a complete geologic record and considering current environmental factors (i.e., topography, weather patterns). The modelling software chosen is open-source, relatively easy-to-use and requires minimal computational power: LAHARZ (Iverson 1997; Schilling 1998), VolcFlow (Kelfoun and Druitt 2005; Kelfoun and Vallejo Vargas 2016), and TephraProb (Biass et al. 2016).

The hazard maps and analysis of volcanic hazard impacts stemming from an eruption of Mount Meager are an important tool for communication of potential activity at this volcano. Specific stakeholders that benefit from this work include: Squamish-Lillooet Regional District managers, the Líľwat Nation, Emergency Management BC, and Natural Resources Canada (NRCan); they have been engaged from the start of this work in order to understand their needs as end-users of the resource. This assessment can be used to prioritize mitigation strategies and inform a monitoring program, with a greater understanding of the hazard characteristics that could be expected from a future eruption.

### Mount Meager Volcanic Complex

The Mount Meager Volcanic Complex (Mount Meager) is a glacier-clad volcanic massif in south-western British Columbia, 150 km northwest of Vancouver. Several stratovolcano peaks comprise the massif, resulting in a complex topography. Rivers at the base of the complex follow tectonic lineaments trending NE-SW and NW-SE (e.g., Grasby et al. 2020).

Mount Meager is part of the Garibaldi Volcanic Belt (GVB), the northern segment of the Cascadia Subduction Zone (Green et al. 1988; Read 1990; Mullen and Weis 2013; Mullen et al. 2018; Venugopal 2019). Volcanism of the GVB is related to subduction of the northern end of the Juan de Fuca Plate beneath the North American Plate (Green et al. 1988) and is characterized by low eruption rates due to a young and hot subducting plate (Green and Harry 1999; Mullen et al. 2018). Many volcanoes in the GVB exhibit extreme relief due to the combination of high rates of uplift (Farley et al. 2001) and a long history of glacier cover (resulting in glaciovolcanism, reduced rock strength, and glacial scouring); this includes, but is not limited to, interaction with the Cordilleran ice sheet in the Pleistocene (Wilson and Russell 2018).

**Fig. 1:**
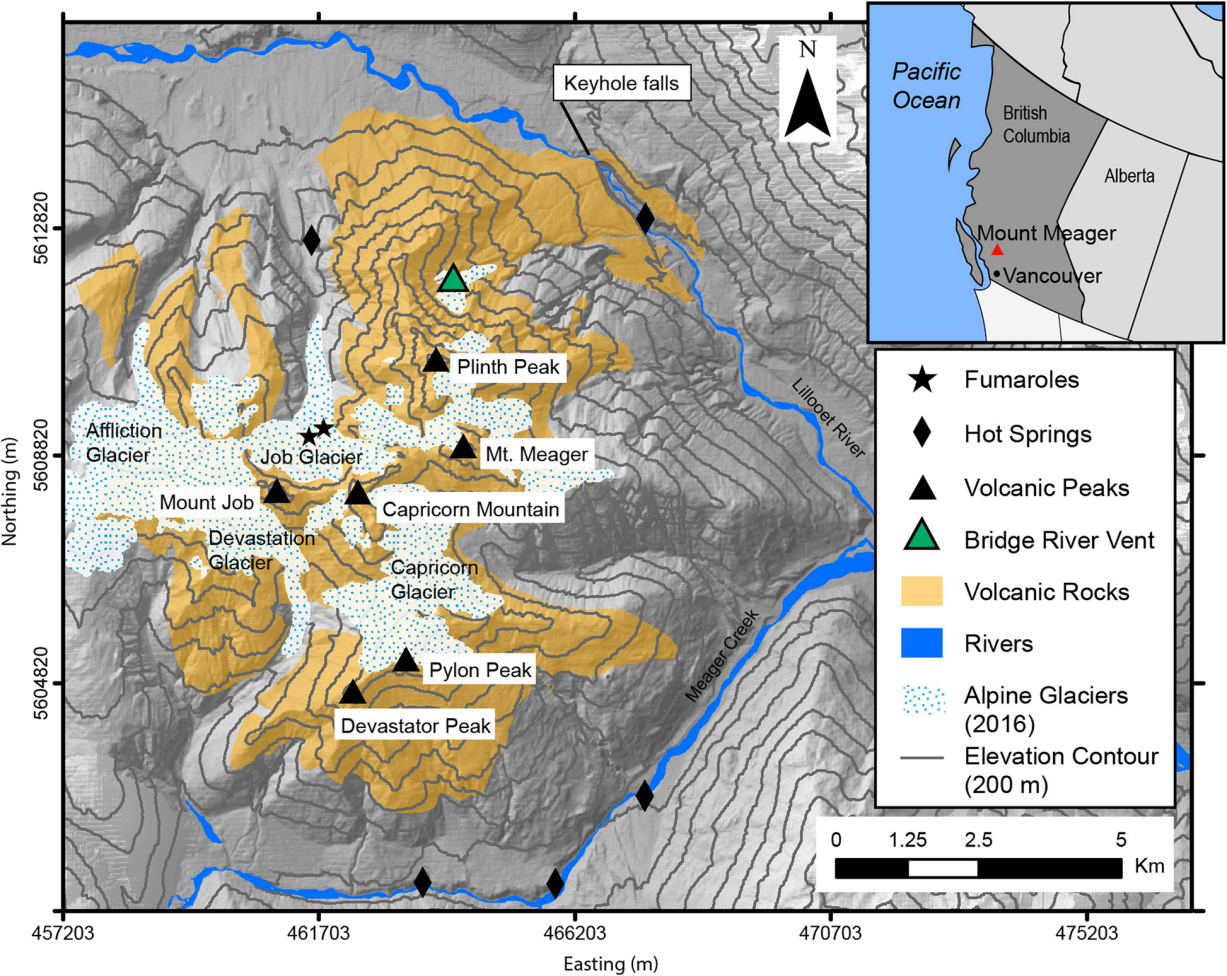
Overview of the Mount Meager Volcanic Complex (MMVC). Includes the extent of alpine glaciers as of 2016, mapped by Roberti et al. (2018). The locations of hot springs (black diamonds) and fumaroles (black stars) represent surface expression of hydrothermal activity. Bridge River Vent (green triangle) is the presumed location of last major eruption. Inset map shows the location of Mount Meager in the context of British Columbia. Base map is composed of a 1 m resolution LiDAR DEM of the massif overlain on 30 m CDEM 092 J and leads to visible discrepancies in base map resolution. The same is the case for subsequent figures. All coordinates are in UTM Zone 10 N, NAD 83

Mount Meager has a 2-million-year history of intermittent effusive and explosive volcanism (e.g., Read 1990; Hickson et al. 1999; Russell et al. 2021) that can be split into three periods: early- and late-stage rhyodacite, and a middle stage of andesitic activity. The eruptive suite includes pyroclastic deposits, overlapping andesite and rhyodacite lavas, and rhyodacite to dacite domes, as well as peripheral basaltic lavas (Read 1990; Hickson et al. 1999). The volcanic deposits are believed to be about 600 m thick and overlie Mesozoic plutonic and metamorphic basement rocks (Read 1990; Roberti 2018). Mount Meager is currently in a state of quiescence; the last eruption occurred 2360 calendar years B.P. (cal yrs. B.P.) from the Bridge River vent (Fig. 1) on the eastern flank of Plinth Peak (e.g., Hickson et al. 1999; Jensen et al. 2019). Current low levels of volcanic activity are manifested by a known high-temperature hydrothermal system, glaciovolcanic caves and fumaroles (e.g., Venugopal et al. 2017; Grasby et al. 2020; Unnsteinsson et al. 2021).

Extensive work by a number of researchers (e.g., Simpson et al. 2006; Friele et al. 2008; Friele 2012) has documented the long record of landslides and established a hazard and risk assessment based on the large-scale and frequent landslides prevalent throughout the complex. Work has continued to document recent mass-wasting events and identify unstable slopes throughout the massif, with volume estimates up to 10^9^ m^3^ (Guthrie et al. 2012; Hetherington 2014; Roberti et al. 2017). Roberti et al. (2018) identified 12 potential landslide sites on the massif with a volume range 10^8^ – 10^9^ m^3^. Only one of the documented debris-flow events has been directly associated with the last eruption (Simpson et al. 2006).

### 2360 B.P. eruption

The most recent eruption to have occurred at Mount Meager (Volcano Explosivity Index, VEI 4; Newhall and Self 1982) is believed to have been episodic; the subsequent emplacement of volcanic products possibly lasted weeks to months, starting as a sub-plinian style eruption and waning to vulcanian style activity (Hickson et al. 1999; Andrews et al. 2014; Campbell et al. 2016).

The vent responsible for the latest eruption is located at 1500 m.a.s.l. elevation, on the northeastern flank of Plinth Peak and is no longer exposed at the surface (Bridge River Vent, Fig. 1). This vent sits atop older volcanic material comprising the Plinth Assemblage, which differs petrographically from the products of the Pebble Creek Formation (PCF) – also referred to as the Bridge River Assemblage in older publications - the deposit from the 2360 cal yr B.P. eruption (e.g., Hickson et al. 1999; Russell et al. 2021). The PCF deposits locally fill the nearby Lillooet Valley. Proximal mapped deposits include fallout pumice, channelized unwelded and welded pyroclastic density currents, volcanic and non-volcanic debris flows, a dacite lava flow and a catastrophic outburst flood deposit (which can also be described as a secondary lahar). Visible tephra fall associated with the eruption has been identified as far away as ~ 550 km E-NE from Mount Meager (Jensen et al. 2019).

The timescale and sequence of events of the 2360 cal yr B.P. eruption have been studied by many researchers (e.g., Stasiuk et al. 1996; Hickson et al. 1999; Stewart et al. 2003; Michol et al. 2008; Andrews et al. 2014). While the explosive phase of the eruption may have only lasted a few hours to a few days, the emplacement of subsequent deposits may have lasted for weeks to months after the initial highly explosive phase. These subsequent deposits include non-welded ignimbrite (indicative of eruption column collapse, or diminished energy in the eruption column) emplaced during or immediately preceding the cessation of violent explosive activity. Welded block and ash flows (BAF), with an estimated volume of 0.15 km^3^, stem from the repeated collapse of lava domes being extruded onto the oversteepened flank of Plinth Peak; welding is due to thick deposits accumulating in the Lillooet River valley at a particularly narrow segment of the terrain (see Keyhole Falls, Fig. 1). Based on mapping by Hickson et al. (1999), the welded BAF at Mount Meager can be traced 5.5 km downstream from the inferred vent location. The extrusion of lava domes and flows could have lasted days to months after the onset of eruption. These deposits are overlain by a non-welded block and ash flow deposit that accumulated in the Lillooet River valley over 1–2 months (Andrews et al. 2014). The welded BAF formed a 110 m high dam directly below the vent which impounded the river and created a lake that subsequently failed catastrophically. The collapse of the dam lead to rapid draining of the temporary lake, leaving behind hyperconcentrated volcaniclastic flood deposits, identified at the base of Mount Meager, and correlated to deposits 45–60 km downstream (Friele et al. 2005; Friele et al. 2008; Andrews et al. 2014). This flood, a secondary volcanic hazard, has been estimated to have occurred over the course of 4 h, depositing 5 × 10^7^ m^3^ of material (Andrews et al. 2014). The final phase of active eruption is represented by a single dacitic lava deposit on the flanks of Plinth Peak with a thickness of 15 m to 20 m (Stasiuk et al. 1996; Hickson et al. 1999). Field observations show that it does not extend to the base of Mount Meager in the Lillooet Valley.

Tephra deposits trends 63° east-northeast and calculations by Hickson et al. (1999) following the Carey and Sparks method (1986) suggest the height of the eruption column was 15–20 km, although it is noted this estimate may be low. A timescale for tephra deposition has not been estimated for this particular eruption.

## Methodology

In this section, the simulation of individual volcanic hazards is described, along with an explanation of the three scenarios that form the basis of this hazard assessment. Each volcanic process has different physical parameters and therefore requires different inputs and numerical models for their simulation (Table 1). These inputs are largely based on parameters of analogous volcanoes in order to overcome the limited geologic data available for Mount Meager. The analogous systems chosen here consist of glacier-clad stratovolcanoes in a subduction zone setting with well documented eruptions and eruption parameters suitable for simulating the hazards at Mount Meager. These include: the 2015 eruption of Cotopaxi, Ecuador (Scenario 1; Global Volcanism Program 2016); the 1985 eruption of Nevado del Ruiz, Columbia (Scenario 2; Pierson et al. 1990) and the 1980 Mt. St. Helens eruption (Scenario 3; Wolfe and Pierson 1995). Although no analogue volcano can be a perfect match and individual eruptions encompass complexities that may not match a plausible event at Mount Meager, given our simple criteria, they are nevertheless deemed suitable. Further explanation of additional source parameters is included within each volcanic process section when data from the reference eruptions did not meet parameter requirements. Given that stratovolcanoes of similar composition to Mount Meager have exhibited a range of explosivities both across their known geologic history and within a single eruptive episode (i.e., Mt. St. Helens, Wolfe and Pierson 1995; Cotopaxi, Bernard et al. 2016), the governing scenarios (Table 1) are guided by parameters consistent with the relative scale of each scenario, capturing a plausible range of explosivity and inundation for future eruptive activity.

**Table 1:** Input parameters for simulated volcanic hazards

Code	Parameters	Scenario 1	Scenario 2	Scenario 3
*Pyroclastic Density Currents*				
Energy cone model, implemented within LAHARZ	∆H/L	0.4	0.3	0.2
	Simulated volume	1 × 10^5^ m^3^	1 × 10^6^ m^3^	1 × 10^7^ m^3^
*Lahars*				
LAHARZ	Reference eruption	Cotopaxi 2015 (Global Volcanism Program 2016)	Nevado del Ruiz 1985 (Pierson et al. 1990)	Mt. St. Helens 1980 (Wolfe and Pierson 1995)
	Reference volume	50, 000 m^3^	1.6 × 10^7^ m^3^	1 × 10^8^ m^3^
	Simulated volumes	1 × 10^6^ m^3^	1 × 10^7^ m^3^	1 × 10^8^ m^3^
	H/L	0.3	0.3	0.3
	DEM resolution	20 m	20 m	20 m
VolcFlow	Coefficient of turbulence	N/A	0.01	0.01
	Viscosity		0.01 Pa.s	0.01 Pa.s
	Density		1600 kg/m^3^	1600 kg/m^3^
	Cohesion		500 Pa	500 Pa
	Internal and basal angles of friction		0°	0°
	Discharge rate		47,000 m^3^/s	47,000 m^3^/s
	Maximum reference flow duration		3 h	4 h
	DEM resolution		20–40 m	20–100 m
*Tephra fall*				
TephraProb	VEI	3	3–4	> 5
	Grid resolution (m)	1000	2500	2500
	Plume height (km asl)	10–15	15–20	20–40
	Erupted Mass (kg)	1 × 10^8^ – 1 × 10^11^	1 × 10^9^–1 × 10^12^	5 × 10^11^–5 × 10^13^
	TGSD range (Ф)	-6 – 8	-5 – 9	-4 – 10
	Median diameter (Ф)	-3 – 0	−1 – 2	0–4
	Sorting (Ф)	1–2	2.5	3–4
	Aggregation coefficient	0.3–0.7	0.3–0.7	0.3–0.7
	Lithic density (km/m^3^)	2600	2400	2200
	Pumice density (kg/m^3^)	1000	700	500
	Diffusion coefficient (m/s^2^)	10	500	2500
	Fall-time threshold (s)	50	500	6000
	Eruption duration (hours)	2–4	4–10	12–120

**Fig. 2:**
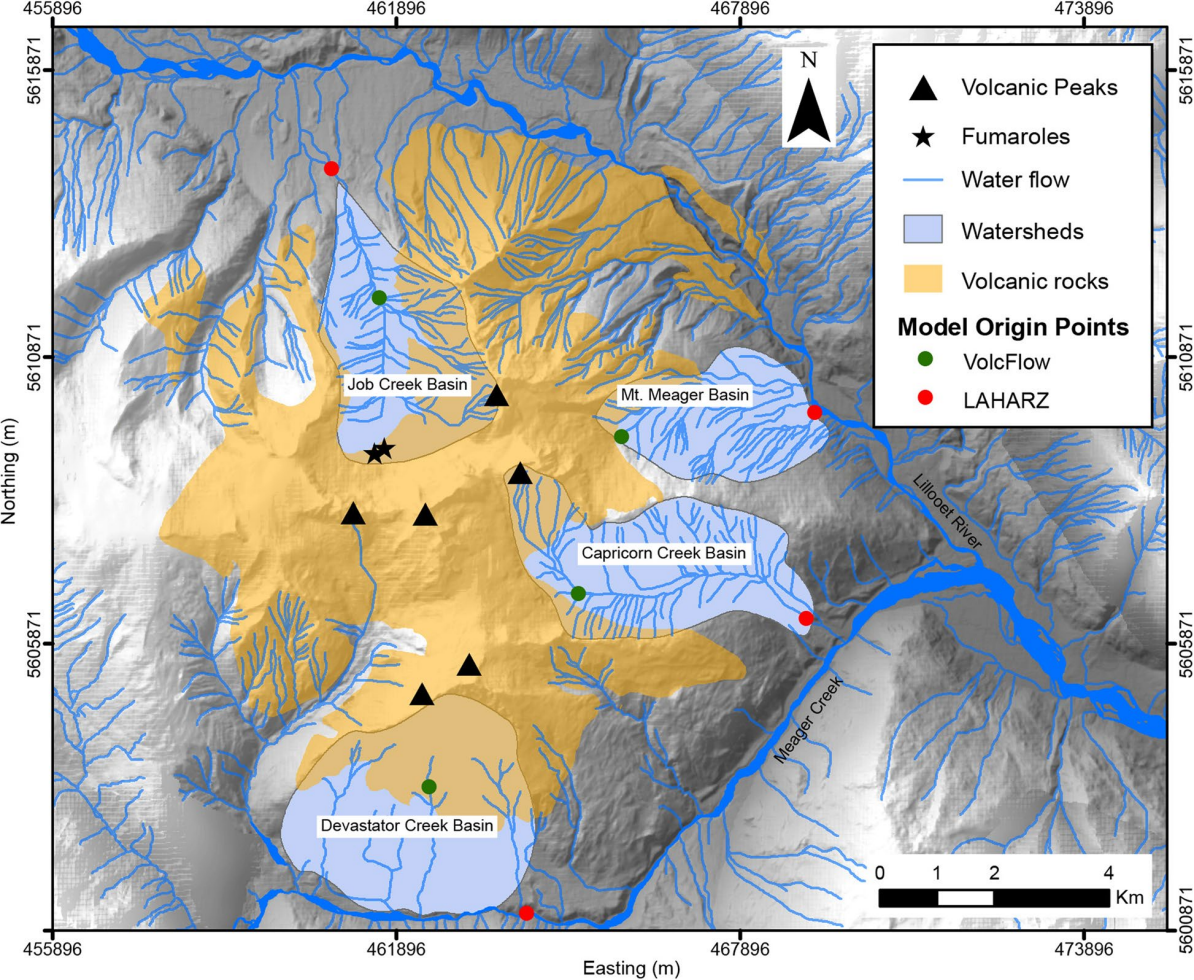
Drainage basins captured by lahar modelling with LAHARZ and VolcFlow. Origin points of each program differed and are represented by green and red dots. All coordinates in UTM Zone 10 N, NAD 83

The three hazard scenarios (Table 1) considered here are separated by eruptive magnitude for the tephra fall hazard, and volume for the dome-collapse PDC and lahar hazards. They are defined (based on value of initial input conditions) as small (scenario 1), medium (scenario 2), and large (scenario 3) magnitude of tephra fall and/or volume of PDCs and lahars. This was done in order to capture the range of plausible eruptive parameters and spatial inundation. The separation of scenarios for the tephra fall hazard follows the Volcano Explosivity Index (VEI) which encompasses eruptive magnitude (force) and volume of erupted material (Newhall and Self 1982). Although an appropriate basis for the tephra hazard scenarios, it is not directly related in practice to the size of a dome-collapse PDC or lahar, which have additional parameters that may influence their size and region of hazard footprint (e.g., Kataoka et al. 2018). As such, the dome-collapse PDC and lahar hazard scenarios are separated by their relative volume. These scenarios do not describe the succession of events that could occur during the onset of an eruption. Furthermore, simulations of lahar propagation and PDC hazards have been carried out from several origin points (four drainage basins, Fig. 2), designed to capture the full scope of the area that could be impacted. At this time, we do not know where the next vent would most likely be located.

The digital elevation models (DEMs), representing topographical surface, used for volcanic process simulation in LAHARZ and VolcFlow were derived from LiDAR (light detecting and ranging) and SRTM (Shuttle Radar Topography Mission). LiDAR was only available for the massif itself, acquired in 2015 (southern half of massif) and 2016 (northern half), at 1 m resolution (Roberti et al. 2018), but downsampled for efficient and timely simulation processing. SRTM included topography beyond the central massif, at 30 m resolution (Farr and Kobrick 2000).

For the following individual hazard maps and combined scenario hazard maps, a distinct colour scheme is followed. This colour scheme is: orange for PDCs, green for lahars and yellow (transitions to blue for individual hazard analysis) for tephra. In this case, the colour scheme does not represent the degree of hazard of one volcanic process over the other. Furthermore, sharp boundaries show the extent of inundation of all individual hazards within their scenario guidelines. However, the degree of hazard does not change abruptly at these boundaries. The degree of hazard decreases gradually with distance from the volcano. Precise hazard zone boundaries are unattainable due to simulation uncertainty and other characteristics.

### Pyroclastic density currents

The energy cone model (Sheridan 1979; Malin and Sheridan 1982) is well-recognized and widely used for pyroclastic density current (PDC) runout forecasting (Ogburn and Calder 2017; and references therein). It is an empirical model based on a statistical analysis that forecasts runout length and incorporates the relationship of the ∆H/L ratio vs. flow volume (Sheridan 1979). This approach requires data on the maximum difference in height from origin of the PDC to the height of its deposit (ΔH) and horizontal length of PDC runout (L). This method depicts the outer extent of PDCs as an energy line (or fully encompassing cone) and incorporates both the dilute and concentrated segments of a pyroclastic density current. The energy cone refers to the area of the slope where frictional loss is balanced by conversion of potential to kinetic energy (Sheridan 1979). This method does not inherently model channelization or directionality but rather covers all interfluves within the energy cone (which may or may not be inundated by overbank PDCs or ash-cloud surges in a real dome-collapse event) (Ogburn and Calder 2017). The LAHARZ package contains an implementation of the energy cone model (called Proximal Hazard Zone Boundary) (Schilling 1998) and was used in this study as it offers a rapid approximation of the runout distance.

Given the very limited information for Mount Meager, the values of the ∆H/L ratio were determined from designated volumes of input based on ∆H/L vs. volume regressions from the global FlowDat database as compiled and formulated by Ogburn (2016, 2012). The regression equations chosen for this work incorporate only BAF data, simulating a dome collapse. The origin points are the apexes of known volcanic peaks (black triangles in Fig. 2).

### Lahars

The literature (e.g., Waythomas 2014; Vallance and Iverson 2015) suggests that primary large-volume lahars are often associated with glacier-clad volcanoes such as Mount Meager. Specifically, these primary lahars are considered to be due to pyroclastic material mixing and rapidly melting glacial ice and snow (Vallance and Iverson 2015). Calculations for Job Glacier, one of the glaciers on Mount Meager with current fumarolic activity, estimate the volume of the glacier to be ~ 7.5 × 10^7^ m^3^ (Farinotti et al. 2019; Warwick 2020) which could contribute meltwater to a lahar stemming from this drainage basin. Melt contribution from a glacier ultimately will not include the entire calculated volume of the glacier as it is unlikely the entire glacial mass would instantly melt and be incorporated into the lahar. However, this value provides a real-world parameter for maximum ice melt contributing to the fluid component of any geophysical flow. Furthermore, Roberti et al. (2018) identified 12 potential landslide areas on the massif with a volume range of 10^8^–10^9^ m^3^. These are not necessarily currently associated with volcanic activity but represent volumes of debris available for failure. Historically, large debris flows initiated from the flanks of Mount Meager have reached up to 10^9^ m^3^ in volume (Friele et al. 2008). While these debris flows are not necessarily syneruptive, these multiple large volume debris flows give a volume range of material that could be incorporated into a lahar associated with a future eruption from Mount Meager.

We chose the LAHARZ code (Iverson et al. 1998; Schilling 1998), a semi-empirically based code, to simulate impact areas that could be attributed to lahars stemming from future volcanic activity. A second geophysical-based numerical code, VolcFlow (Kelfoun and Druitt 2005; Kelfoun and Vallejo Vargas 2016) was used in this study for further analysis of impact area in addition to assessment of flow time and thickness values. Simulations were only carried out for governing scenarios 2 and 3 with VolcFlow (all three are covered by LAHARZ simulations).

Processing in LAHARZ requires just a few steps. First, a stream grid is created (representing the surface hydrologic network). Next, the slope of the energy cone is defined, which represents the separation between the lahar erosive area and accumulation area. The intersection of this boundary with a hydrologic line marks an origin point for lahar simulation. Finally, volumes of inundation are specified. In this case, volume increments defining each scenario (Table 1) are consistent with the logarithmic scaling of LAHARZ. The program output constructs a nested set of inundation-hazard zones, with each zone representing the user-specified volume of input (Iverson et al. 1998).

Simulations were tested and run on both the available LiDAR DEM and SRTM DEM. The LiDAR was downsampled to 5 m resolution for use with LAHARZ. Simulations run on the SRTM DEM (30 m resolution) were carried out where the runout distance reached beyond the area covered by the LiDAR DEM (i.e., scenario 2 and 3). A value of 0.4 was used for the ∆H/L ratio. This value produced a boundary line across all drainages that roughly corresponds with the mouth of each drainage, which in this case is assumed as the point at which lahar deposition starts. Following the steps of running LAHARZ, four points of simulation origin were chosen (at the intersection of energy cone boundary to hydrologic grid stream-line). This was done in order to capture lahar propagation stemming from key drainage basins (Devastator Creek, Capricorn Creek, Mt. Meager and Job Creek basin) – encompassing drainage from south, east and northern flanks of the complex (see Fig. 2). The output nested inundation footprints often exhibit unrealistic lateral inundation areas in the form of ragged edges (Iverson et al. 1998; Muñoz-Salinas et al. 2009). The final modelled outputs in this study show the boundaries of lahar inundation as smooth lines for display purposes. In the case of hazard mapping at Mount Meager, a few metres of inaccuracy as a result of smoothing the boundaries should not impede future interpretation and hazard management due to the remoteness of the area.

VolcFlow simulates gravitational flows and captures the mass flow behaviour in time and space (Kelfoun and Druitt 2005; Kelfoun and Vallejo Vargas 2016). It has been applied to debris avalanches, dilute and concentrated components of pyroclastic density currents, landslide derived tsunamis, lava flows (Kelfoun and Vallejo Vargas 2016), and lahars (Gueugneau 2014; Vasconez et al. 2017). Flows are modelled using depth-averaged equations of mass and momentum conservation on a topography linked coordinate system where x and y parallel the local topography. VolcFlow solves for several types of rheological equations (e.g., frictional, viscous, or plastic) (Kelfoun and Druitt 2005; Kelfoun and Vallejo Vargas 2016). This feature is manifested by the choice of the retarding stress parameter, which is user-defined by manipulating the value of cohesion. Other input parameters also affect the physical flow characteristics including: internal and basal friction angle, viscosity and a dimensionless parameter that defines turbulent or collisional stress. The single-layer model has been used here for the lahar modelling.

The LiDAR DEM was used for the VolcFlow simulations with a resolution downsampled to improve model runtimes; coarse resolutions (60 m – 100 m) were chosen to set up the simulations and test efficiency and final runs were on DEMs with 20 m - 100 m resolution. The physical input parameters were largely informed by two previous studies, i.e., Gueugneau (2014) efforts modelling the Armero disaster from the Nevado del Ruiz eruption in 1985, and the reconstruction of lahars from the 1698 eruption of Carihuairazo, Ecuador (Vasconez et al. 2017). The input parameters remain the same for each drainage basin and differ only by initial volume for each scenario.

Simulation of origin points started midway down the slope. In the case of the Job Creek basin, this location is generally placed at the terminus of the glacier as of 2016 observations. For the other three locations, the initiation points are located at the boundary of the volcanic massif and its underlying basement rock. The equations behind VolcFlow solve for momentum and mass balance, starting at these points, and capture both the failure of dominantly volcanic material and the momentum achieved on each of the slopes. Discharge is modelled from a point source, as a single pulse, by setting the program to add mass at the designated discharge rate until total initial volume has been reached. The simulation is stopped when maximum velocity is consistently low (below 3 m/s).

With this iteration of modelling, the physical parameters of the flow remained constant, which is a simplification of the lahar flow dynamics as it does not account for bulking and debulking behavior. In particular, the interaction with additional fluid components is not included, such as the natural river discharge present in the area.

### Tephra fall

The only component of probabilistic hazard analysis within the scope of this study was accomplished from modelling tephra fall using TephraProb. This code is based on the Tephra2 model (Bonadonna et al. 2005) and uses the advection-diffusion equation to determine the tephra mass accumulation given varying eruption scenarios differing by eruption and wind conditions. It incorporates a total grain-size distribution (TGSD) file to account for particle aggregation processes. During computation, the model samples input source parameters (see Table 1) for each run (making it a probabilistic assessment). For this study, the sampling of plume height is logarithmic and both plume height and TGSD integration steps are set to 50. The number of runs was set at 100. The Eruption Range Scenarios (ERS) function was implemented, stochastically sampling eruption source parameters and wind profile (Bonadonna et al. 2005; Biass et al. 2016). The NOAA Reanalysis 1 dataset was chosen (Kalnay et al. 1996), accessed through the graphical user interface for wind input parameters (see Biass et al. 2016 for additional information). This dataset provides 4 wind profiles per day for the years chosen (for this study, 2006 to 2011).

The thresholds of concern that have been applied to data produced for the Mount Meager simulations are: 1 kg/m^2^ to account for road functionality implications and minor impact on agriculture, 10 kg/m^2^ to account for electric failure, minor roof damage, and crop damage, and 100 kg/m^2^ which can lead to roof/building damage (Biass et al. 2017; Blake et al. 2017; Wilson et al. 2017). All parameters are taken from reported values in the literature. This includes the aggregation coefficient, maximum aggregation diameter, diffusion coefficient and total grainsize distribution (Biass et al. 2016), and fall time threshold (Bonadonna et al. 2005; Biass et al. 2016). Lithic and pumice density are based on density ranges reported by Scollo et al. (2008).

## Individual hazard maps

In the following section, the outcome of each hazard simulation is described, and individual hazard maps visualize the extent of inundation that could occur from each modelled process.

### Pyroclastic density currents

**Fig. 3:**
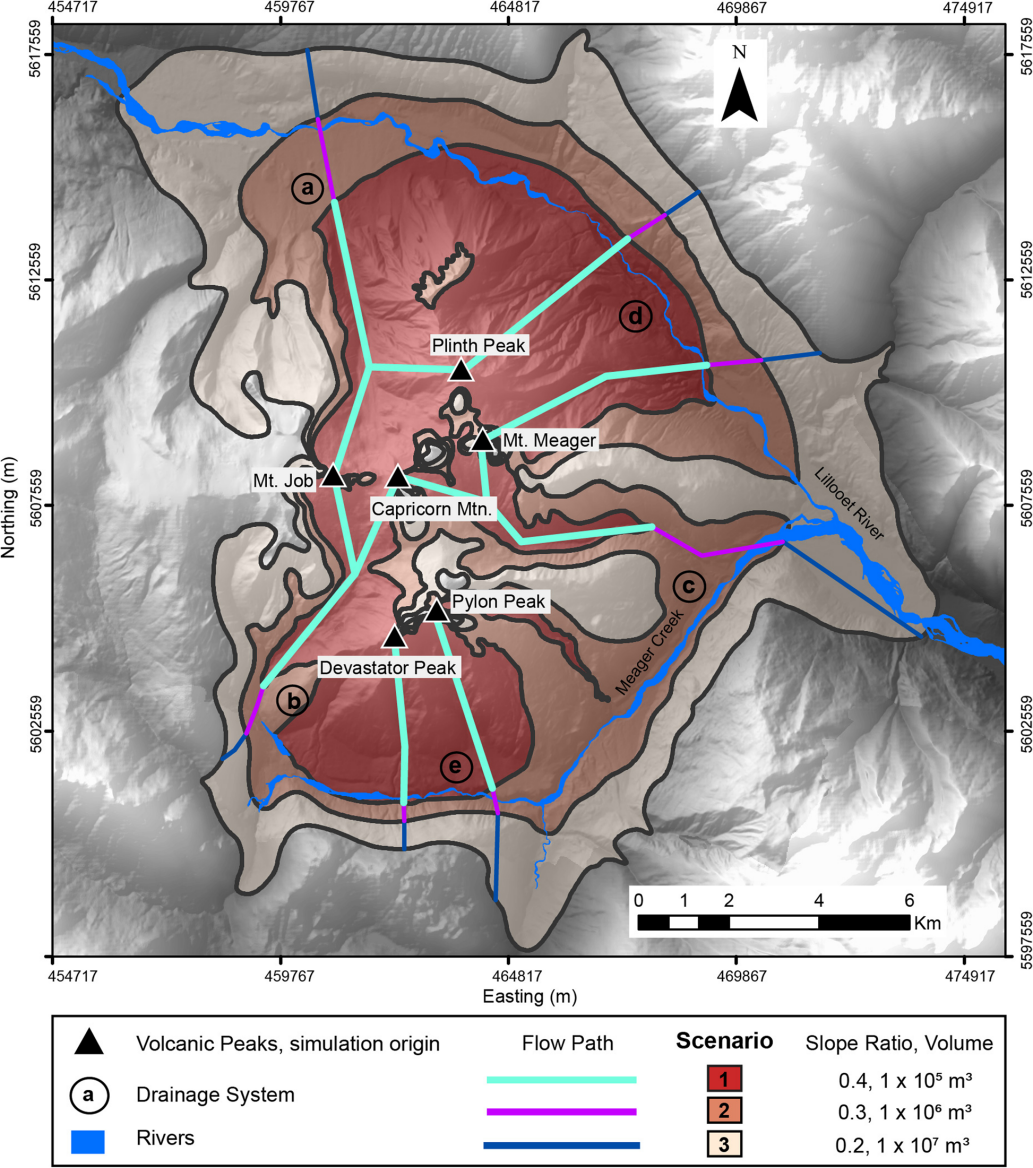
Combined results of dome-collapse PDC scenarios modelled with the energy cone model. The shaded relief displays the possible areas that could be impacted from PDC events given the input parameters selected for a dome collapse PDC event. The coloured lines (cyan for scenario 1, pink for scenario 2 and blue for scenario 3) represent the line where the runout length extent measurement was made, based on planar measurements. The letters (**a**-**e**) categorize the drainage systems as described in Table 2. All coordinates are in UTM Zone 10 N, NAD 83

Pyroclastic density current (PDC) modelling with the energy cone model produces zones of hazard, separated by boundaries that represent a simulated maximum runout length in accordance with the input parameters. Shaded regions (Fig. 3) represent areas that may be inundated by ash-cloud surges or be an overestimation of spatial coverage as this method does not explicitly model channelization. For this reason, we have added lines on the PDC hazard map (Fig. 3) that show our approximation of the direction and maximum runout path (Table 2) that could occur in the event of a PDC being generated from current volcano apexes within the massif. These represent a selection of significant channels within the massif, based on topography.

**Table 2:** Maximum simulated runout length (km) of pyroclastic density currents modelled by the energy cone model from volcano apex to hazard zone boundary

Drainage system	Scenario 1	Scenario 2	Scenario 3
*Mt. Job*			
a	6.2	8.1	9.6
b	5.4	6.5	7.3
*Plinth Peak*			
a	5.6	7.5	9.0
d	4.7	5.6	6.5
*Mt. Meager*			
d	5.3	6.5	7.7
c	5.3	8.4	12.2
*Capricorn Mountain*			
c	6.0	9.1	12.8
b	5.5	6.6	7.4
*Pylon Peak*			
e	4.0	4.5	6.5
*Devastator Peak*			
e	3.5	4.0	4.6

The simulations for scenario 1 (representing a dense basal avalanche volume of 1 × 10^5^ m^3^) show that PDCs could reach distances of 3.5–6.2 km. Meager Creek may be inundated from a PDC stemming from Devastator Peak, and the Lillooet River could be inundated from a PDC stemming from Plinth Peak and Mt. Meager. Scenario 2 simulations (volume 1 × 10^6^ m^3^) show that maximum runout lengths could reach 4.0–9.1 km. Simulations of a scenario 3 (volume 1 × 10^7^ m^3^) eruptive event could generate PDC runout distances of 4.6–12.8 km. Both Meager Creek and Lillooet River would be subsequently inundated from a PDC stemming from any volcanic apex given scenario 2 and 3 parameters. Based on the volcanic peaks used as starting points, the PDC hazard does not encompass the entire massif - the western edge is absent of inundation from this hazard.

### Lahar

**Fig. 4:**
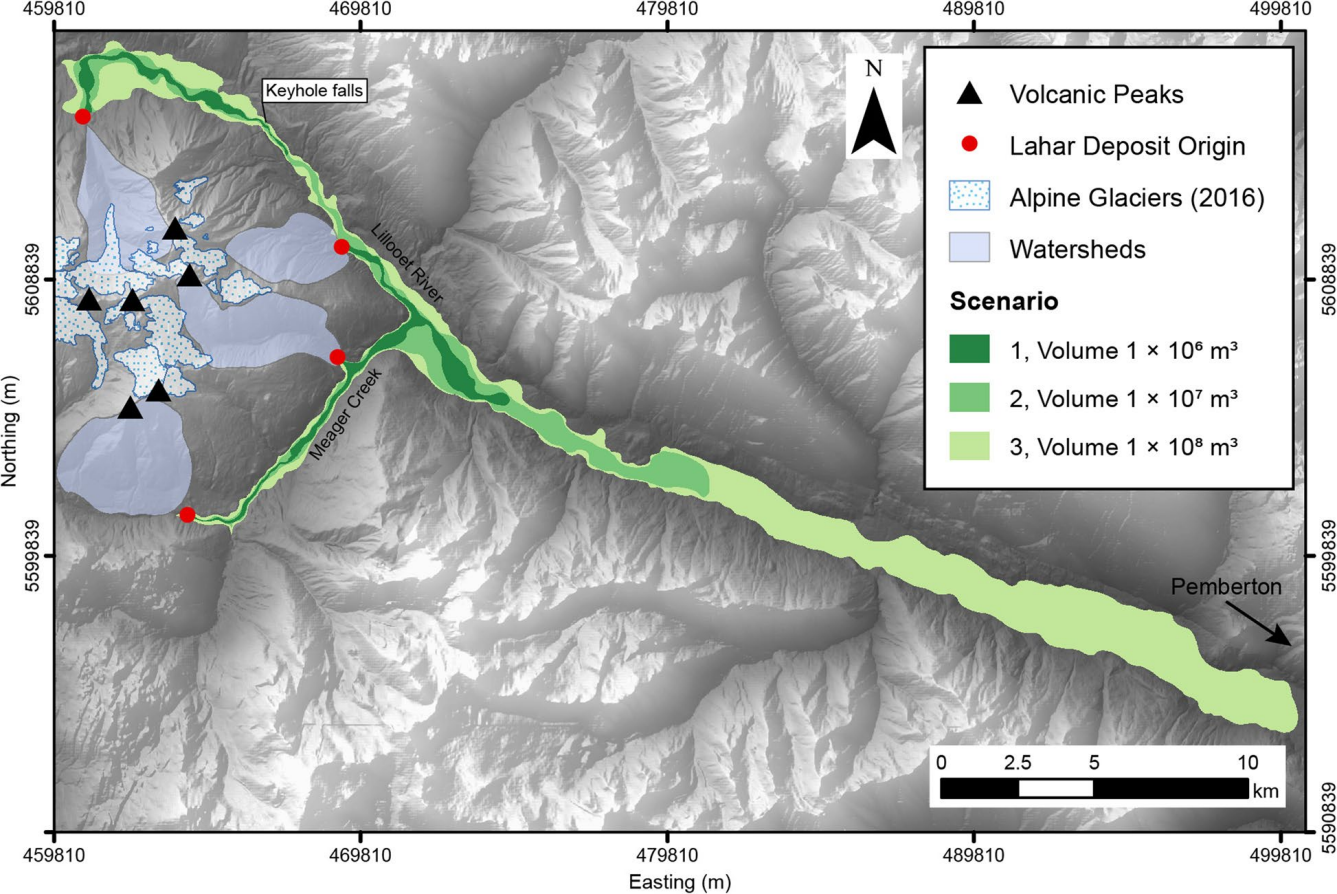
Scenario-based lahar flow hazard map modelled by LAHARZ. The spatial runout is presented for three scenarios, differentiated by flow volume input. They represent the governing explosive eruption scenarios for a possible future eruption from Mount Meager. The red dots indicate the start points in LAHARZ which represent points where erosion ends, and lahar deposition begins. All coordinates are in UTM Zone 10 N, NAD 83

The potential lahar inundation zones generated by LAHARZ are presented in Fig. 4. Each lahar scenario presented on the map represents the merged volume and the simulated flows are intended to represent primary flow hazards and are considered as single pulse events (Schilling 1998).

Table 3 lists the individual results of each scenario from the four separate points of origin. Runout length includes the length from point of origin to the end of the modelled flow (horizontal planar distance). Inundation area includes total planimetric area covered by the separate flow scenario. The area was calculated from unedited shapefiles generated in LAHARZ, which include unrealistic jagged edges; the “jagged edges” have been smoothed in subsequent maps.

**Table 3:** Summary of quantitative output values from modelled lahar inundation with LAHARZ

	Scenario 1	Scenario 2	Scenario 3
Source (Easting, Northing, Elev., m)	Devastator Peak drainage basin (464165, 5601197, 686)		
Run out length (km)	11.5	20.1	39.9
Inundation area (km^2^)	2	9	43
Source (Easting, Northing, Elev., m)	Job Creek drainage basin (460780, 5614204, 809)		
Run out length (km)	9.6	21.5	40.6
Inundation area (km^2^)	2	9	43
Source (Easting, Northing, Elev., m)	Mt. Meager drainage basin (469180, 5609902, 440)		
Run out length (km)	7.6	14.5	34.4
Inundation area (km^2^)	2	9	43
Source (Easting, Northing, Elev., m)	Capricorn Creek drainage basin (469052, 5606262, 584)		
Runout length (km)	6.6	13.4	33.4
Inundation area (km^2^)	2	9	43

Runout lengths vary while inundation area does not as operationally, the planimetric area is pre-determined based on the scaling arguments of LAHARZ. The volume parameter remains the same across all scenarios and therefore, the planimetric area will be the same for all equivalent scenarios.

The lengths of single-event lahar flows are predicted as: 6.6 km – 11.5 km (scenario 1), 13.4 km – 21.5 km (scenario 2), and 33.4 km – 40.6 km (scenario 3). All simulated flow paths are directed towards terrain of lower topography, with all eventually reaching the Lillooet River, either directly (Job Creek and Mt. Meager flows) or indirectly (Devastator Peak and Capricorn Creek flows inundate Meager Creek first before entering Lillooet River).

**Fig. 5:**
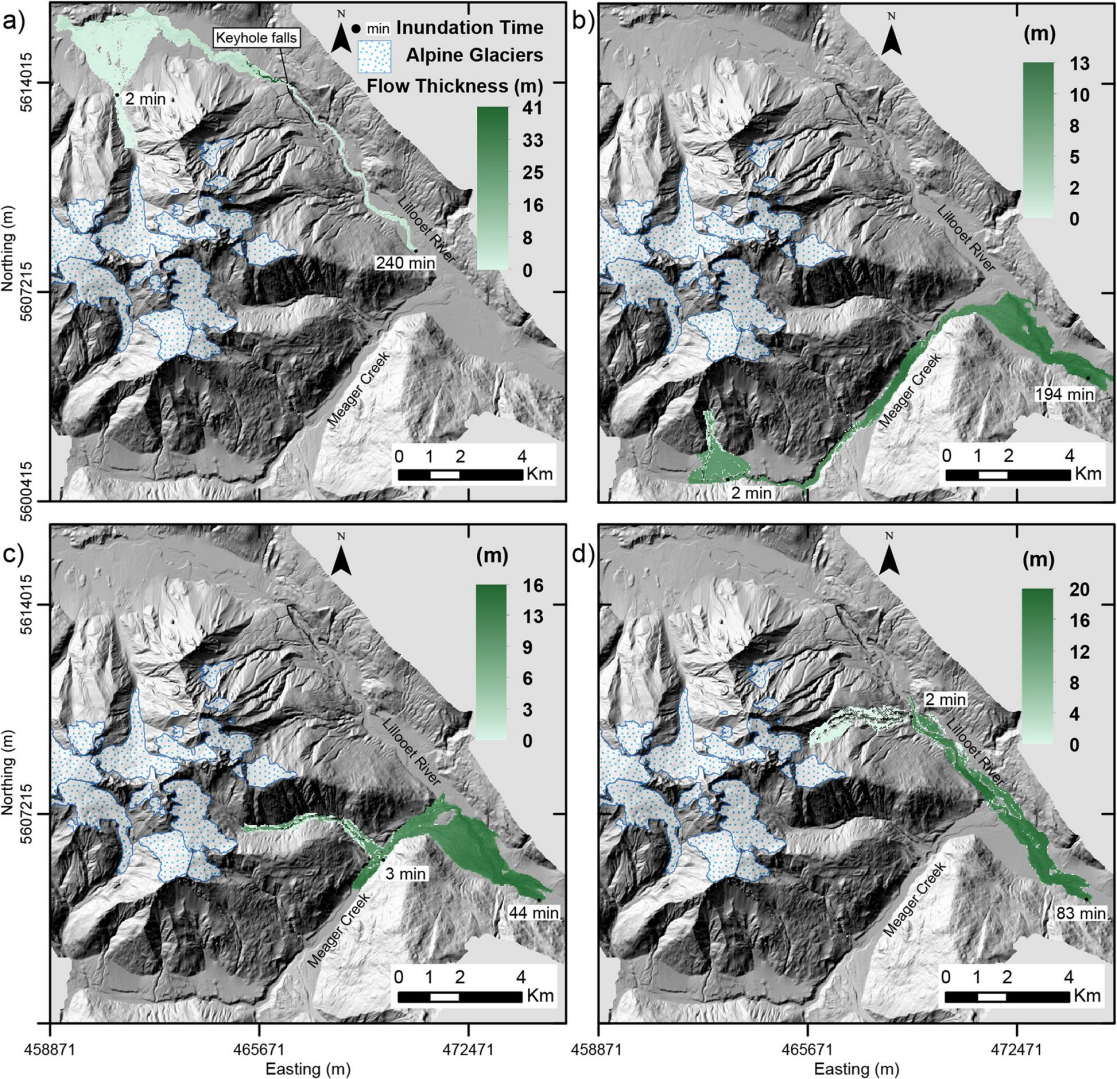
Scenario 2 lahar simulation results (inundation footprint, thickness and time) modelled in VolcFlow for lahars stemming from **a**) Job Creek, **b**) Devastation Peak, **c**) Capricorn Creek and **d**) Mt. Meager. All time stamps are approximate, and the gradient of thickness differs across all simulations. All coordinates are in UTM Zone 10 N, NAD 83

**Fig. 6:**
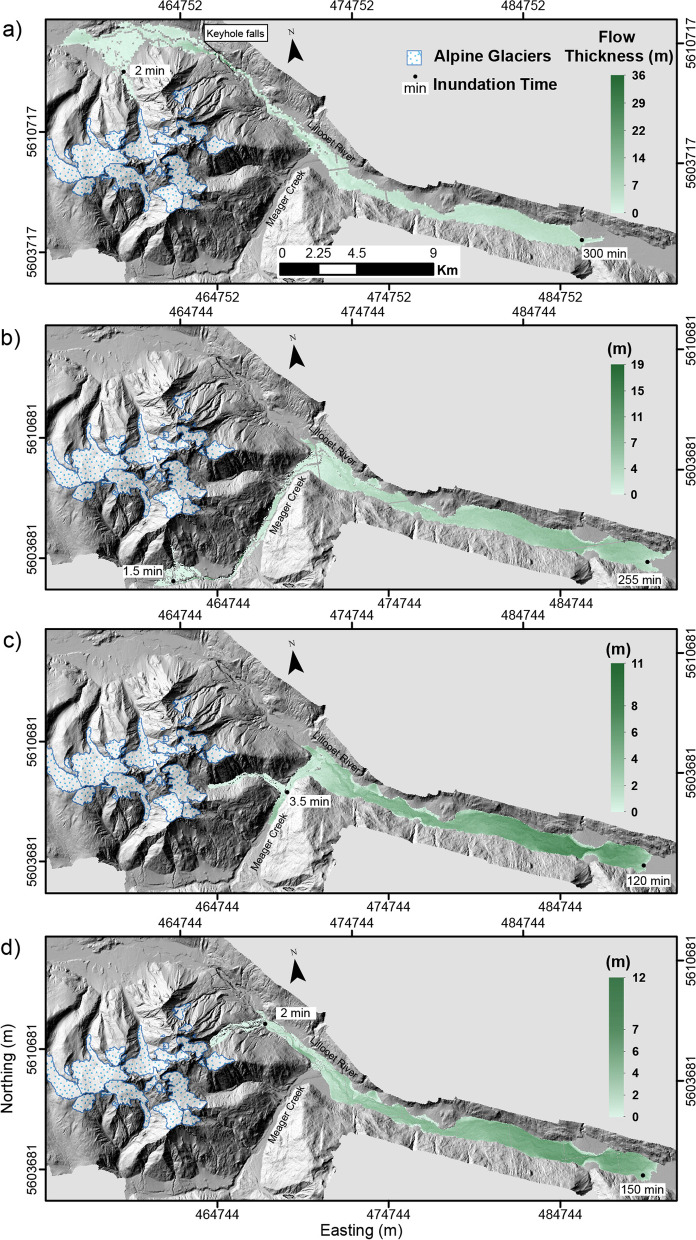
Scenario 3 lahar simulation results (inundation footprint, thickness, and time) modelled in VolcFlow for lahars stemming from **a**) Job Creek, **b**) Devastation Peak, **c**) Capricorn Creek and **d**) Mt. Meager. All time stamps are approximate, and the gradient of thickness differs across all simulations. All coordinates are in UTM Zone 10 N, NAD 83

Simulating lahars with VolcFlow rendered an inundation footprint (area and flow length), flow thickness and flow time. A standard deviation value is ascribed to the thickness component, computed with the statistical analysis tool in QGIS. Time stamps are included in Figs. 5 and 6, which report the simulated timeframe of propagation. This is linked to flow velocity which varies across the duration of propagation due to physical parameters such as terrain slope and valley confinement. Additionally, elapsed computational run time (desktop computer) is included (Table 4). Computational run time varied with each scenario and run depending on the DEM resolution (high resolution resulted in longer processing time) and simulation time to reach consistent low maximum velocity, indicating end of simulation.

**Table 4:** Results from lahar simulation modelled in VolcFlow at the end of simulation run time

Basin	Devastation Creek		Capricorn Creek		Mt. Meager		Job Creek	
Scenario	2	3	2	3	2	3	2	3
DEM resolution (m)	20	60	20	20	40	20	20	100
Deposited volume (10^7^ m^3^)	1.0	10.0	1.0	10.0	1.0	10.0	1.0	10.0
Total length (km)	18.2	34.9	11.6	27.8	11.7	29.2	17.9	35.0
Inundation area (km^2^)	6.7	26.4	6.1	24.8	4.7	20.7	6.8	22.4
Simulated time (hours)	4.0	6.0	0.8	2.0	1.5	2.8	4.0	6.0
Elapsed time computational	6 h	50 min	1.6 h	11 h	29 min	11 h	14 h	28 min

In all cases, as expected, flow direction is dominantly directed towards points of lower elevation. For lahars stemming from the southern section of the massif, this results in lahars entering Meager Creek and being directed east into the Lillooet River. For lahars stemming from the northern and eastern slopes of the massif, this results in direct flow and deposition into the Lillooet River. Scenario 2 lahars reach a total length of 11.6 km - 18.2 km and scenario 3 lahars reach a distance of 27.8 km - 35 km.

Deposited scenario 2 lahars are all dominantly less than 2 m thick and scenario 3 lahars less than 4 m. The maximum value of thickness is reached in the Job Creek lahar simulation in both scenario 2 (24–41 m) and 3 (18–36 m), with flow thickness concentrating behind Keyhole Falls, the most confined segment of terrain in the area of Mount Meager. Throughout all simulations, maximum thickness values range from 13 m – 41 m (scenario 2) and 11 m – 36 m (scenario 3). Flow accumulation occurs at segments of natural valley confinement and at the toe of the lahar.

In all simulations (scenario 2 and 3), lahars reaching the major valley bottom (Meager Creek or Lillooet River) occurs in under 3.5 min (100 s – 210 s). Devastator Peak and Capricorn Creek lahar simulations take 8 to 21 min (scenario 2) and 8 min to 27 min (scenario 3) to reach the Lillooet River. The scenario 2 Job Creek lahar is the only simulated flow that does not reach past the confluence of Meager Creek and Lillooet River. At a total inundation time of 4 h, it is simulated to stop along the Lillooet River close to the southeastern corner of the base of the complex (ahead of the confluence). Otherwise, the scenario 3 lahar initiated from Job Creek reaches the point of confluence in 64 min and the scenario 2 and 3 lahar initiated from the Mt. Meager drainage basin reaches this point in under 10 min. The speed of lahars down the flanks of Mount Meager reach 17–30 m/s (similar values in scenario 2 and 3) and slow down with distance from the source and flow over flatter topography.

### Lahar modelling synthesis

It is clear that the present drainage pattern of the area on and surrounding the volcanic complex will dominate the trajectory of any lahar stemming from Mount Meager. The dominant water course at the base of Mount Meager is the Lillooet River, which all modelled flows enter and continue downstream, either directly or indirectly if stemming from the southern sector of the complex. These flows are all constrained within the confines of the existing channel boundaries. The simulations show that lahar deposits for scenario 2 and 3 will inundate the Lillooet River, which would lead to significant damage to existing infrastructure and facilities within and close to the margins of the river.

In comparing the results and operation of LAHARZ and VolcFlow, distinct differences between the two become apparent. LAHARZ is fast and computationally simple, VolcFlow is more powerful with respect to opportunities for investigating the rheological conditions of the flow (thickness, velocity, flow morphology), in addition to the investigation of surface inundation. However, VolcFlow requires more processing power and computing time. In all scenarios, LAHARZ produces longer lahar runout distances than VolcFlow. The differences are on the order of ~ 6 km for equivalent scenario 2 simulations and ~ 10 km for equivalent scenario 3 simulations. VolcFlow was better able to account for constriction points in the topography (narrow valleys), as it uses a depth-averaged approach for simulation, and this would ultimately affect the rheological characteristics. Finally, more data can be extracted and investigated, readily with VolcFlow, including parameters such as propagation time and flow thickness.

### Tephra fall

**Fig. 7:**
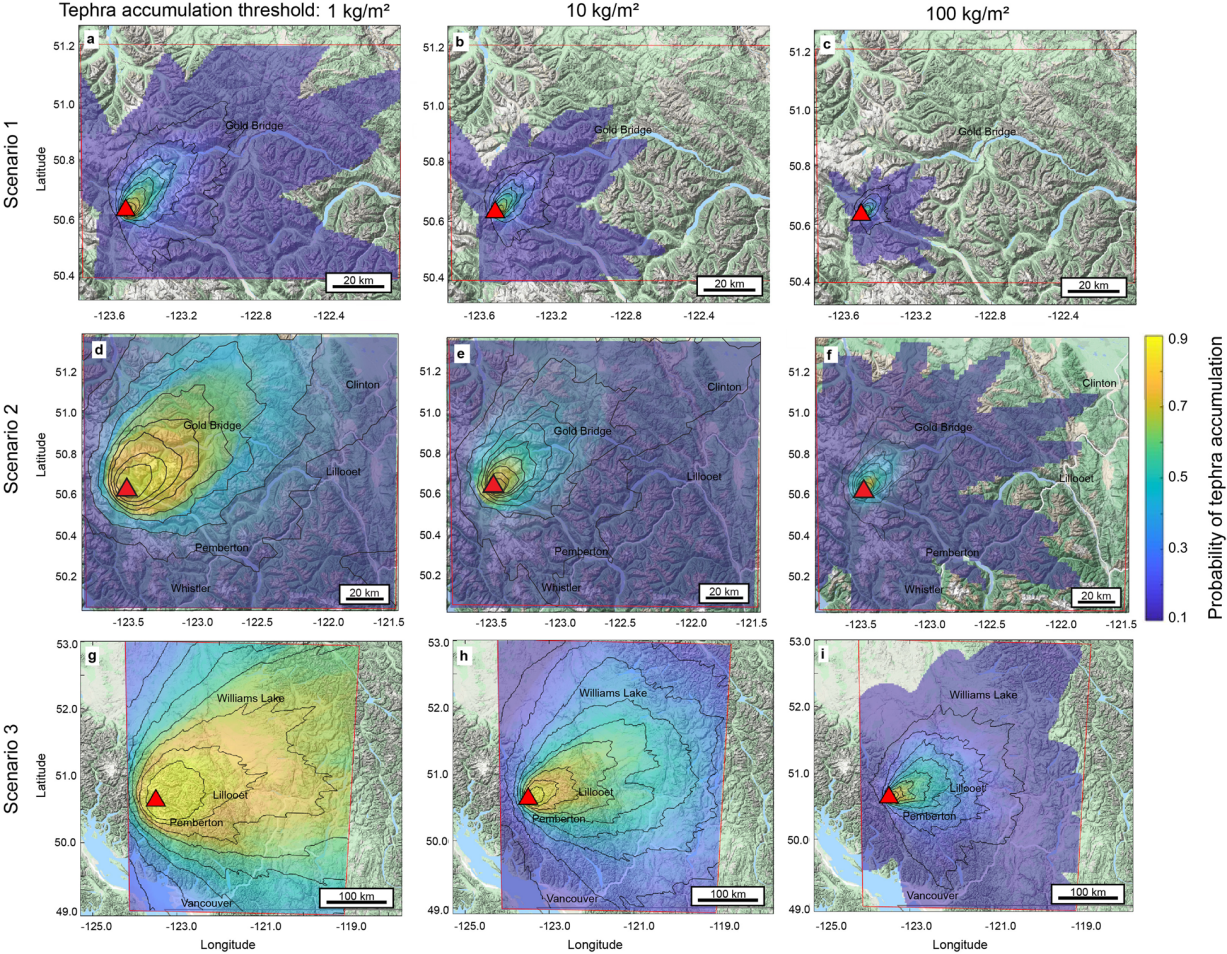
Mount Meager tephra deposition hazard maps for the probability of exceeding a threshold of tephra accumulation conditional on the eruption scenario as modelled by TephraProb. Scenario 1 **a**) for a tephra accumulation of 1 kg/m^2^, **b**) 10 kg/m^2^, **c**) 100 kg/m^2^; Scenario 2 **d**) 1 kg/m^2^, **e**) 10 kg/m^2^, **f**) 100 kg/m^2^; Scenario 3 **g**) 1 kg/m^2^, **h**) 10 kg/m^2^ and **i**) 100 kg/m^2^. Contours indicate the probability starting at 0.1 and incrementally increased by an interval of 0.1. Red line indicates the boundary of the computed grid and the red triangle locates the eruption vent. Coordinates are WGS 1984

The probability of exceeding thresholds of tephra accumulation (1 kg/m^2^, 10 kg/m^2^, and 100 kg/m^2^) are mapped as spatial distributions resulting from modelling with TephraProb (Fig. 7). Results for a few select towns/population centres are presented below (Table 5). With the exception of the Upper Lillooet Recreation Site, the locations included in Table 5 for scenario 1 and 2 include towns that are closest to Mount Meager and within range of being impacted by the simulated tephra hazard. The Upper Lillooet Recreation Site is not a location with any permanent population but it is a proximal landmark. The cities/ towns highlighted in scenario 3 represent notable population centres at incremental distances in the various directions modelled to be affected by tephra fall. These towns are shown as examples and do not include all population centres that may be impacted in the modelled scenarios.

**Table 5:** Results for probability of exceeding the threshold in designated population centres affected by an eruption of Mount Meager

			Exceedance probability		
*Scenario 1*					
Location	Distance	Direction	1 kg/m^2^	10 kg/m^2^	100 kg/m^2^
Gold Bridge	56 km	E	6%	2%	N/A
Upper Lillooet Recreation Site (50.616, − 123.392)	5 km	SE	12%	11%	10%
*Scenario 2*					
Location	Distance	Direction	1 kg/m^2^	10 kg/m^2^	100 kg/m^2^
Gold Bridge	56 km	E	56%	34%	8%
Lillooet	111 km	E	22%	7%	N/A
Pemberton	65 km	S	19%	10%	N/A
*Scenario 3*					
Location	Distance	Direction	1 kg/m^2^	10 kg/m^2^	100 kg/m^2^
Kamloops	222 km	E	72%	47%	8%
Williams Lake	195 km	NE	64%	34%	7%
Pemberton	65 km	S	88%	50%	24%
Vancouver	165 km	S	27%	12%	2%

A clear directionality is present in tephra deposition, that being dominantly directed towards the northeast (Fig. 7). This direction is dominantly 45° NE in scenario 1 and 2 and 65° NE for scenario 3. It is also consistent across all scenarios that the contours are geographically closer to Mount Meager, the origin, when exceeding 100 kg/m^2^ as opposed to 1 kg/m^2^.

The sphere of impact from a scenario 1 eruption is restricted to a proximal area surrounding Mount Meager (Fig. 7a, b, c). Only two locations are noted in Table 5, neither of which have significant permanent populations but are notable landmarks in the vicinity of Mount Meager. The furthest extent of the 10% contour probabilistically exceeding a 10 kg/m^2^ threshold is within a range of 30–55 km from Mount Meager. The Lillooet River has less than 80% probability of exceeding the accumulation of 1 kg/m^2^ of tephra, less than 70% with a threshold of 10 kg/m^2^, and less than 50% probability with a threshold of 100 kg/m^2^.

For scenario 2, modelling shows that the Village of Pemberton has the potential to be impacted by tephra accumulation exceeding the thresholds of 1 kg/m^2^ or 10 kg/m^2^, along with the major waterways and dedicated utility structures for the town.

Pemberton may be impacted by tephra exceeding all specified accumulation thresholds conditional on scenario 3 parameters. Furthermore, Metro Vancouver may also be impacted by tephra accumulation exceeding 1 kg/m^2^ or 10 kg/m^2^ for scenario 3. Finally, Kamloops and Williams Lake are only included in the computational grid for scenario 3 and are therefore not captured within the scope of scenario 2 (Fig. 7).

The uncertainty of contour location, and therefore the location of impact increases with distance from the source from ±3 km to ±10 km. This was determined based on observing the outputs of the runs (where total number of runs was set to 100), where difference in location of the outer contour position varies more than that contour position at the source.

## Scenario hazard maps of Mount Meager explosive eruptions

The individual hazards have been combined as scenario hazard maps below. These indicate areas of impact that could occur from the different phenomena stemming from explosive activity for a range of magnitudes. The occurrence and extent of inundation by each individual hazard is dependent on its individual physical characteristics but discrete hazard areas are combined as scenario hazard maps to exemplify the multi-hazard nature of eruptive events.

Lahar inundation results from LAHARZ and VolcFlow (for scenario 2 and 3, where simulation results were obtained by both programs) were merged within ArcGIS. LAHARZ produced longer lahar runout distances than VolcFlow. The differences are on the order of ~ 6 km for equivalent scenario 2 simulations and ~ 10 km for equivalent scenario 3 simulations. The area of lahar inundation in these hazard maps therefore reflects the flow from the point of presumed origin (in this case considered to be the terminus of the glacier) to the total inundation length considering both programs.

The hazard maps show the extent of each hazard as a solid boundary. In reality, the extent and true coverage of events are not justified by sharp boundaries but are rather gradational boundaries to match their inherent uncertainty. However, with the exception of results from TephraProb, all hazard characteristics were developed from deterministic modelling, which produce artificially abrupt boundaries.

### Scenario 1: small magnitude eruption; small volume lahars and PDCs

**Fig. 8:**
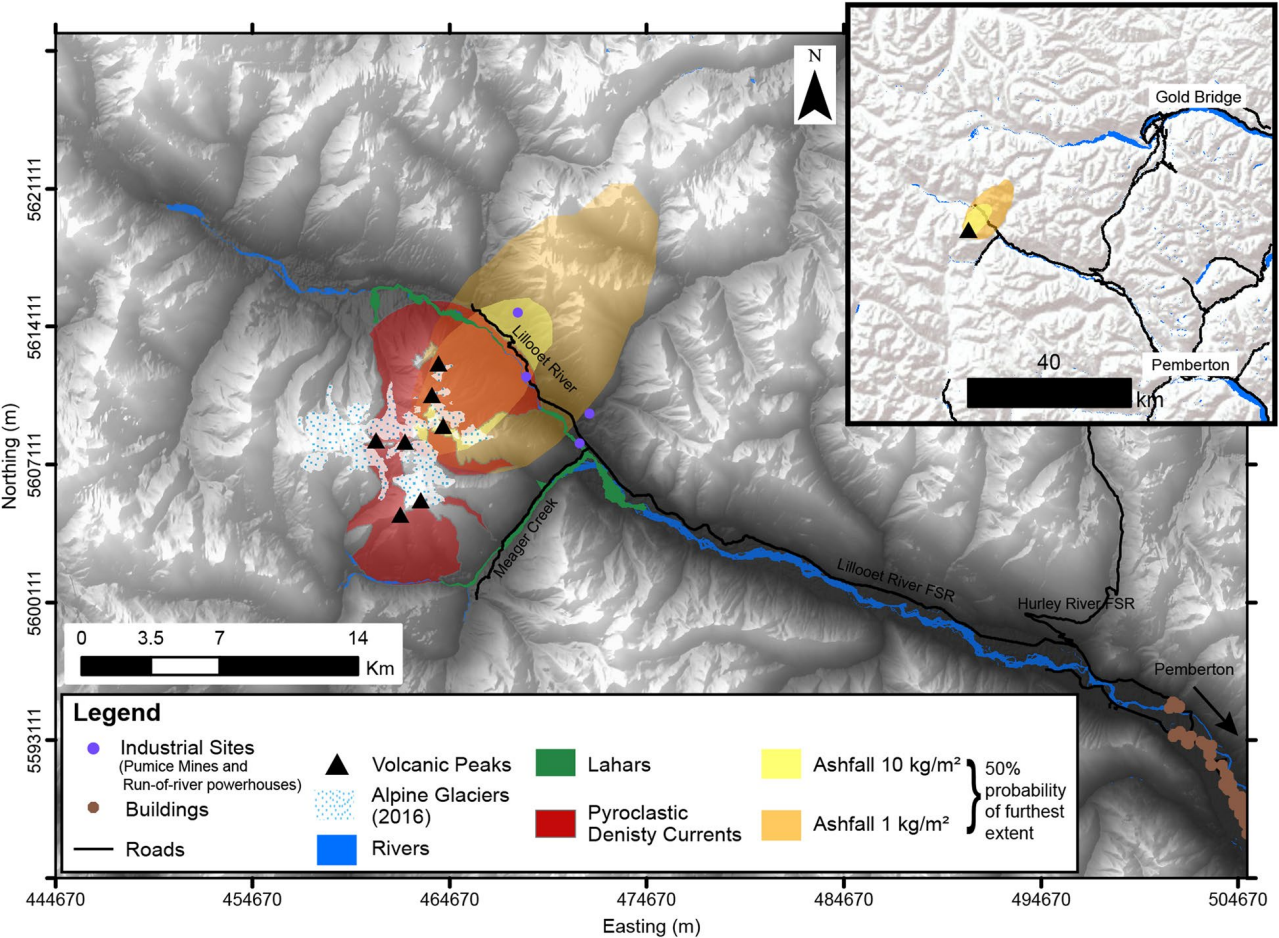
Volcanic hazard map for scenario 1, conditional on the occurrence of a small scale explosive eruption). The upper Lillooet River valley will likely be affected by inundation from lahars, pyroclastic density currents, and tephra fall. Additionally, tephra fall may impact areas beyond the Lillooet valley, with deposition affecting mountain ranges north-east of the complex (map inset). All coordinates are in UTM Zone 10 N, NAD 83

In the case of a small explosive eruption, PDC, lahar and tephra deposition hazards will likely be limited to the nearby vicinity of the volcano (Fig. 8). Pyroclastic density currents could largely be confined to the region of the massif itself, only extending to the base of the complex with eruptions triggered from Plinth Peak, Mt. Meager or Pylon Peak. Lahars could inundate Meager Creek and the Lillooet River up to a maximum runout length of 11.5 km. Tephra accumulation will likely be limited to unpopulated regions around the volcano, with possible implications to industrial activity in the area (such as logging, pumice mining operations, and renewable energy generation). The most widespread accumulation of tephra > 1 kg/m^2^ (1 mm) thickness may be deposited up to 30 km from the volcano (greater than 30% probability). Across all modelled hazards, population centres are not likely to be impacted in this scenario. The areas of impact will probably be restricted to the massif itself and, most importantly, the river systems that surround the volcano.

### Scenario 2: mid-magnitude eruption; medium volume PDCs and lahars

**Fig. 9:**
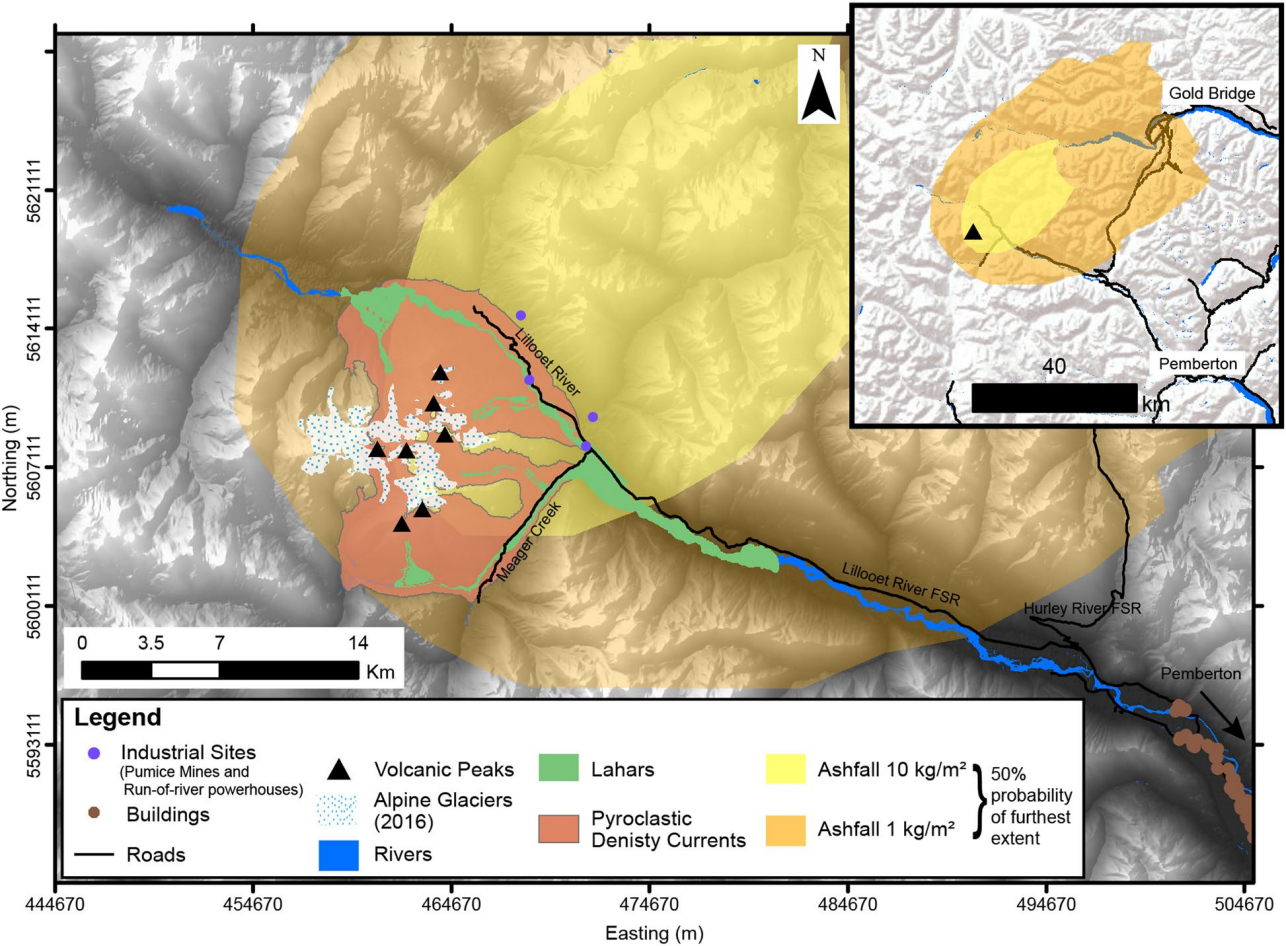
Volcanic hazard map for scenario 2, conditional on the occurrence of a mid-scale eruption. Areas that could be affected by lahars and PDCs include an extension of the upper Lillooet River valley and PDCs covering the river channels (Meager Creek and Lillooet River). Tephra fall may fully encompass the massif and affect areas up to 90 km NE (50% probability), see inset map. All coordinates are in UTM Zone 10 N, NAD 83

The areas that could be impacted by a mid-range explosive eruption exceed the base of the volcanic complex (Fig. 9). PDC deposits will most likely inundate the main river system (individual simulations initiated from Mt. Job and Capricorn Mountain do not), extending inundation up to ~ 2.5 km beyond the base of the complex. This distance covers roads and other industrial infrastructure within the upper Lillooet River valley. Lahars will likely follow the course of Meager Creek and the Lillooet River up to a maximum runout length of 21.5 km, and simulations show they may follow the natural path of the river system, flowing towards (but not reaching) the inhabited region of Pemberton Meadows. Simulations show it could take between 2 to 3 min from the point of initiation to reach the main waterways that discharge into Meager Creek or the Lillooet River, although this timing is dependent on the point of initial elevation and therefore distance travelled to the main river system. Tephra fall of all modelled tephra accumulation thresholds will likely be restricted to southwestern British Columbia, with a low probability of impacting any major population centres. TephraProb results restrict the furthest extent of impact exceeding 1 kg/m^2^ to within 100 km from the volcano which only includes the community of Gold Bridge, a small service centre for nearby recreation-residential properties. The region, with a probability of impact greater than 30%, also includes a section of Pemberton Meadows, an important agricultural area. This amount of tephra accumulation has implications for road functionality and creates potential for limited crop damage (Blake et al. 2017; Wilson et al. 2017).

### Scenario 3: large magnitude eruption; large volume PDCs and lahars

**Fig. 10:**
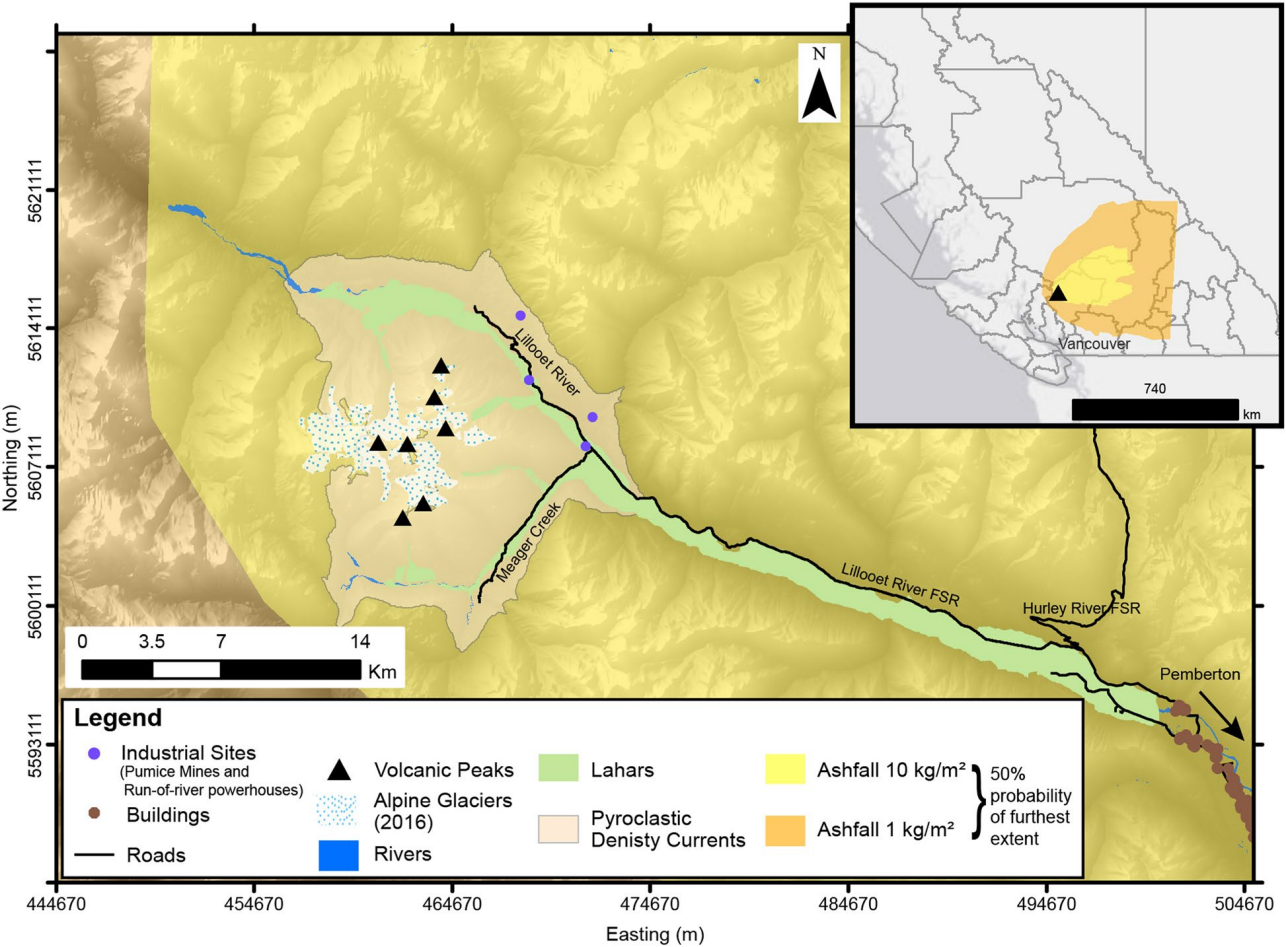
Volcanic hazard map for scenario 3, conditional on the occurrence of a large scale eruption. The upper Lillooet River valley will likely be affected by all simulated hazards. Pemberton Meadows will also likely be affected by lahars and tephra fall. The furthest extent of tephra fall (50% probability) may impact areas of southwest British Columbia (see map inset). All coordinates are in UTM Zone 10 N, NAD 83

In the case of a large explosive eruption, similar to the 2360 cal yr B.P. eruption of Mount Meager, inundation from the volcanic hazards could far exceed the perimeter of the massif, impacting infrastructure, agriculture and residential properties (Fig. 10). As a proximal hazard, pyroclastic density currents would still likely be confined to the region immediately surrounding the volcanic complex, extending 5 to 6 km beyond the base of the complex, to opposing mountain sides up to an elevation of ~ 1000 m. The potential zone of inundation includes the Lillooet River, Meager Creek, logging roads, industrial infrastructure and recreational sites within the vicinity of Mount Meager. Lahars could travel up to ~ 30 km beyond the edge of the massif down the Lillooet River valley (this instance occurs from flows stemming from the south-eastern area of the complex). This total distance of inundation could include Pemberton Meadows, covering about 8 km^2^ of agricultural land. The total inundation distance may cover significantly more of the logging and forest service roads beyond those in the scenario 1 and 2 lahar inundation. The two main forest service roads that would be impacted and rendered inaccessible are the North and South Lillooet Forest Service Road and Hurley River Forest Service Road. This in turn would cut off road access to facilities in the vicinity of Mount Meager, including those used by logging operations, pumice mining and the run-of-river hydro project on the Upper Lillooet River. Popular back-country recreation sites accessed by these roads would also become inaccessible by road.

The grid chosen for the spatial probability calculation in TephraProb only extends to ~ 350 km from the volcano. At this limit, TephraProb calculates the probability of exceeding 1 kg/m^2^ mass load to be greater than 60%. Within this degree and greater probability, population centres such as Kelowna, Kamloops, Williams Lake, Pemberton and surrounding smaller communities and land would be impacted. In this scenario, Metro Vancouver and the Lower Mainland have a 30–40% probability of being impacted by an exceedance of 1 kg/m^2^ tephra mass load.

## Discussion and conclusion

Volcanic hazard assessments are important in preparing for, managing, and mitigating the impacts of volcanic eruptions. They are commonly developed for well-known volcanoes with geologically recent or ongoing eruptive episodes. However, there is a distinct lack of hazard assessments produced for systems that are seemingly remote, and characterized as low eruptive frequency, but that may in fact be high impact (Wilson and Kelman 2021). This could be attributed to limited resources and time available for studies on these volcanoes. However, with improved computer codes (many of which are available as open source), and recognition of the validity of numerical modelling as a mapping tool for hazard impact studies, the ability to produce hazard assessments on these systems is more feasible and efficient. Mount Meager is an excellent example of a low eruptive frequency system with the possibility of high impact (presumed from geological mapping of the 2360 cal yrs. B.P. eruption).

The key outcomes of this study demonstrate that:

- A scenario-based approach captures a range of volcanic hazard phenomena that could be reasonably expected from future eruptive episodes;
- Appropriate numerical models, using parameters from well-studied analogous volcanoes, can supplement the known geologic record and effectively overcome limited evidence of past eruptive history;
- A future eruption at Mount Meager can generate a multi-hazard event, impacting areas as close as 5 km to where a vent may open, and up to several hundred kilometres away, given a large magnitude eruption (>VEI 5).

The hazard assessment here represents a first iteration of volcanic hazard mapping for Mount Meager. Limitations of this study exist, and it may be improved or expanded upon in the future. For example, only syneruptive primary volcanic hazards associated with explosive eruptions were considered, with three hazard phenomena chosen to be modelled and spatially analyzed.

Other hazards could certainly result from an eruption, syn-eruptively or post-eruptively. These could include growth/movement of the fumarole field within Job Glacier or extending to other parts of the glacier system, with implications for hazardous volcanic gases. The stability of the glacier is also in question with collapse of glaciovolcanic caves due to degassing and melting being highly likely; this hazard needs to be considered when people visit the glacier. Lava flows are also likely features of the next phase of volcanism. These features may only be restricted to the confines of volcano slopes themselves due to the petrological characteristics of the system (dacitic/andesitic composition) and therefore have minimal direct implications to human activity in the vicinity of Mount Meager. They will, however, alter topography, possibly changing the course of the simulated hazards presented here. The failure of lava flow fronts, in addition to dome collapse, were the cause and instigation of PDCs during the last major eruption (Stasiuk et al. 1996), and have implications for the extent of the PDCs hazard considered here. Secondary lahars (e.g., rain-triggered) may occur for an indefinite time period following an eruption. As such, lahars are extremely unpredictable and mitigation necessitates the installation of a lahar detection and alerting system that monitors the arrival and passage of mass movements, similar in function to systems in place around Mount Rainier, Washington (Pierce County 2008; National Park Service 2021). To be effective, such a system would require a significant effort by emergency management agencies to develop and exercise response plans for detection of potentially hazardous lahars, and to prepare the public for such events. Debris flows, whether considered as separate slope failure events or related to the volcano itself, are known hazards of Mount Meager (e.g., Simpson et al. 2006; Roberti et al. 2018).

While this study showcases the development of a hazard assessment for a remote volcanic system with limited knowledge of eruption history, this assessment would benefit from further geological investigation of the massif. This would allow us to use parameters specific to Mount Meager for better modelling which would, no doubt, improve the assessment. This assessment is limited by the fact that only explosive eruption hazards are considered, but the geological record does suggest periods of effusive activity have occurred throughout the complex (Read 1990). Future work should include the modelling and assessment of effusive eruption hazards (such as lava flows) either aided by further geological investigation of the complex or following the framework of this study. This would aid in the development of a more comprehensive volcanic hazard assessment for Mount Meager.

Finally, this hazard assessment is well-suited to inform the development of a monitoring network (e.g., regular gas and geochemical sampling of fumaroles and hot springs, automated detection system for mass movement, dedicated seismic network, analysis of Interferometric Synthetic Aperture Radar (InSAR) images to monitor ground deformation). This would be of immediate benefit for ongoing human activity in the vicinity of the volcano.

This hazard assessment is an important step towards the development of a robust and reliable Disaster Risk Reduction strategy that should be developed for Mount Meager and other potentially hazardous volcanic systems throughout Canada. A generalized version of the three scenario hazard maps will be published as an open-file report by the Geological Survey of Canada. This serves to publicize the data and findings of the assessment to a broader audience of interested citizens in Canada.

## Data Availability

Data access information and additional methodologies are provided in the manuscript or in supplementary materials.
